# Town Smoke and Fogs

**Published:** 1888-10-27

**Authors:** David Walsh


### Town Smoke and Fogs.

By David Walsh, L.R.C.P. and S., Edin.

There is poetry in a smoke wreath, there is picturesqueness in the blue-grey eddies that fade away into thin air. Yet the poets who sing and the painters who sketch these beauties are alike dealing with what is in sober truth a messenger of disease and death. It is of the cities that one speaks thus, and anyone who climbs a church tower or other high place overlooking the sea of roof-tops, will hardly fail to be struck with the countless rows and stacks of chimneys always ready to add their burden to the smoke-laden atmosphere. At a rough guess there are about as many chimneys as inhabitants in a large town. In the poorer parts, where a family often lives in one room, there will be many people to its single fireplace, but on the other hand may be placed the factories and public buildings, besides the mansions of the rich, carrying, as a rule, several chimney-pots to each member of the household. Those who study the processes of life, tell us that each citizen is a kind of machine, breathing off the products of combustion from his lungs night and day, and in a certain broad and general sense every house may be said to do the same thing through its chimneys. There is this difference, however, that what is thrown off from the lungs is clear and made up of comparatively simple chemical substances, while the visible smoke from fires contains in addition soot and other things which are the result of imperfect combustion.

There is much truth in the old saying, "more fire, less smoke," and speaking generally it may be stated that if the combustion of coal were perfect there would be no smoke, for almost all the fuel would be converted into water, carbonic acid, and small quantities of other equally colourless gases. But before that stage is reached, we have what are known as "intermediate products," tar, oils, soot, and other things that would quite disappear if mixed with a certain quantity of air and burnt. Common smoke may be shortly defined as the visible fumes of burning fuel, although a wider use of the term would include the invisible products as well. The yellowish flame of a common gas jet does not smoke so far as can be seen, yet it is nevertheless loaded with solid and half-burnt substances. Everyone knows that an earthenware plate held over a gas flame soon gets covered with a layer of lamp-black. That is the carbon or soot of imperfect combustion, which is being constantly poured forth from the gas-burners of our rooms and houses. Pass the same gas, however, through a Bunsen's burner, which mixes it with air before burning, and note the difference. The flame is no longer yellow, but burns with a beautiful lambent blue or purple colour, like that of a spirit lamp; it gives off the most intense heat, and no longer deposits lamp-black. In short, it is the flame of perfect combustion, although unsuitable for ordinary purposes, since the illuminating power of a Bunsen's burner is small. But the fact remains that the coal-gas with which we are in the habit of lighting our rooms, is only half-burnt, and pours out soot and damaging invisible products, besides using up a large amount of oxygen. Coal-gas lighting may therefore be termed a wasteful process; it is hurtful in many ways, both to health and to property, and unworthy of the present scientific age. Much the same line of reasoning applies to the use of coal as a source of warmth. Electricity has none of the drawbacks of ordinary gas for lighting, as it neither consumes oxygen nor gives off harmful products, and when ways and means are bettered, there is little doubt we shall turn to that quarter as the great future source of artificial light and heat.

Public health workers will find in this question a pretty field for reform. All the smoke—one hears them say—curling away from the housetops; the black clouds vomited from the factory chimneys; the fumings of the locomotives; nay, the very braziers of the plumber and the hot-potato vendor; all are at one in this great conspiracy to poison our town air with the products of imperfect combustion. And their indictment of the nuisance would be wanting, unless every gas-jet, every oil-lamp, and every candle were included. In all these, the chief smoke element is the unburnt solid carbon or soot. It is the one which concerns us most in the present paper, dealing as it does with the open-air aspect of smoke pollution, but a word may be said with advantage of some of the other products. Leaving out of consideration carburetted hydrogen, which is the main source of the flame, that part of the carbon which does not come off as soot unites with the oxygen of the air, to form a part of the invisible smoke, carbonic acid gas, and which ceases to be injurious when mixed with plenty of air. A certain amount of water is formed by the union of hydrogen and oxygen, and this is of course harmless. The sulphur, of which a small quantity exists in all coal, may come off in various forms. Combined with oxygen, it constitutes the colourless sulphurous acid, which is said to act as a general antiseptic or purifier of the atmosphere; or it may come off as sulphuretted hydrogen, an inflammable gas. Travellers by the underground railways are familiar enough with the sulphurous fumes from the coal or coke of the engines. There are other products about which much might be written, but as they have little bearing on the health question, we shall pass on to the consideration of the main element, carbon. This is carried up the flues by the currents of heated air from the fires, and is found in several states of division. It may be in the form of finest dust, like the lamp-black collected on a plate from a flame that seems to be smokeless, or it may be deposited in the chimneys as "soot," and when this catches fire it almost looks like a revengeful and satirical effort on the part of the flues to show how easily perfect combustion can be attained. Or the carbon may float about in the little flakes that everyone knows as the "blacks." These are the smuts that settle so persistently on our faces and on our clothes; that drift in at open windows, and are ruinous to white curtains or delicate wall-papers; that are the bane of the laundress, and the terror of all good housewives. Look at the plants on the town window-sill, and you will see the leaves of the geranium or ivy thickly dusted with the all-pervading soot-flakes. It is, of course, a well-known fact that plant life will not flourish in cities, and the chief difference between the air of town and country lies in the smoke. Sheffield, her atmosphere grimy and soot-laden from countless fires, would at present hardly nurture a blade of grass; but there is no reason why plants like the harebell and the dog-rose should not thrive in its streets if by chance at any time they were to become deserted and smokeless. Where plants thus suffer animals do not escape.

Among affections of the lung is a group known as the "dust diseases," due to the presence of inhaled particles of foreign matter. For a long time it had been known that workers in certain trades, such as millers, coal miners, stonemasons, and steel grinders, were very subject to consumption, but it is only of late years that medical men have begun to understand the relation of the dust to the disease. All modern observers of any note are pretty well agreed that consumption is mainly due to the presence of tiny living organisms. These "bacilli," as they are called, will not thrive in healthy lungs, and it is only in damaged tissues or cells that they can get a hold. Dust sets up inflammations of various kinds in the air-passages, and opens the door to the bacilli. Were these organisms able to attack healthy lung few people could hope to escape, for the air must be charged with enormous numbers of them in a climate cursed with consumption, like that of Great Britain.

The lung of a sheep is a familiar object in a butcher's shop, where it is commonly known as the "lights." It is a spongy structure of a delicate pink colour, and since the animal has lived in smokeless country air, is quite free from specks of carbon. Compare with this the lung of a man who has lived an open-air life in the Hebrides. The two are very much alike, both are pink and spotless; indeed, a Shetlander's lung is taken by the lecturers on medicine as a type of what that organ should be. But now go to a museum and look out the lung of a Londoner, and you will find a structure grey and full of black spots and patches, which are in fact all due to the soot from the fires of the vast city in which his life has been passed. Lastly, turn to the lung of a collier, which will be dark grey or black, for the substance of it is filled with the actual coal-dust of his underground workings, until it has become almost like a sponge full of ink. Here then are three degrees between the positive of health and the superlative of disease. First, the Shetlander's lung, which is clear and bright; next, the town-dweller's, grey and mottled; and thirdly, the collier's, which is black, baleful, and blighted. After this, no one will be surprised to hear that the average length of life and tendency to consumption stand in exactly the same order. The chances are that the Shetlander will die of old age, the town-dweller somewhere about middle age, and the coal-miner before his prime. The transformation of the particles gives the citizen a decided advantage over the collier, for the coal-dust of the mine is far more irritating than soot, but both are alike foreign bodies when breathed into the air-cells and air-tubes. Some interesting sums might be worked out by the statistical societies as to how much of the coal carried every year into the metropolis is taken to the cemeteries in the lungs of the Londoners. But apart from curious speculations comes the important question as to whether the evil cannot be lessened, or altogether prevented. In the light of recent progress, and the fuller recognition of the danger, one may venture to predict that some day—much to the benefit of our crowded communities—the air of cities will be no longer polluted by smoke. A suggestive lesson can be gathered from a chapter in the history of knife-grinding in Sheffield. Under the old system the use of the dry grindstone was the rule, so that the workshops were filled with the dust of stone and steel, and it was common for the grinders to die of consumption at thirty. Remonstrances, either with masters or men, were of no avail, and at length Government interfered, and dry grinding was made a punishable offence. Wet grindstones then became the order of the day, with the effect that what had before been carried into the air as dust was washed down into the troughs as a fine sand, and the lives of the workmen are now lengthened up to the average in spite of themselves. The remark is often heard that people cannot be made moral by Act of Parliament, but here one sees an instance of how legislation can, at any rate, make them healthy.

Just as grindstone dust was got rid of by improved methods, so the reformer may not unreasonably hope that an equal amount of good may be effected some day in smoke prevention. Much must be left to the weight of enlightened social opinion—much more to the progress of electrical and other sciences, which will replace the present clumsy and carbon-producing methods of obtaining artificial light and heat. Government has already curtailed the manufacturer's right to pollute whole districts by compelling him to consume his own smoke, and by allowing for a few minutes only each day the black clouds that follow on firing-up his furnaces. The principle of almost all smoke-consuming or purifying furnaces is to procure perfect combustion by mixing with air before burning, just as in a Bunsen's burner. All the soot is burnt along with products that would otherwise be wasted, so that a great saving is effected in fuel. The vast problem, however, of how to deal with household fires has yet to be solved. Good has been done by the adoption of anthracite or "smokeless" coal, by slow-combustion grates and stoves, by the substitution of gas, and by the use of better methods of heating with hot air or hot water. Your Englishman, however, is a creature of habit, and it will be long ere he forsakes his dear old homely, but wofully unscientific, fireside: perhaps not before his coal-fields are exhausted, and there is no longer the wherewithal to fill the scuttle. He cannot be said to have advanced his method much since the days when the smoke was allowed to wind among the rafters and escape through a hole in the roof: that hole is now longer and more elaborate, but the wasteful, soot-producing fireplace is still much the same. Let us hope that some mechanical genius will one day invent the wished-for grate—smokeless, cheap, and efficient. Such a man would add to the health and hence to the happiness of his fellows to an extent not readily appreciated, and would deserve a niche in the Temple of Fame along with the inventors of the steam engine and the electric telegraph.

To some of our readers this may seem like over-statement, but let them think for a moment how the abatement of smoke would revolutionise the whole surroundings of life in cities. The purity of the air would allow plants and animals an equal chance of flourishing in the town as in the hamlet. The Londoner's lung would become as clear and—other things being equal—as resistant to the attacks of the tubercle bacilli as that of the Shetlander, and so would tend to strengthen the stock of our communities. But there are other questions of social economy involved besides that of health. There is the saving of fuel, already alluded to, and then there are the fogs, which have been shown to depend to a great extent on the presence in the air of finely-divided dust. It is interesting to note in passing that the words "foggy" and "muggy" are derived from allied roots. The actual loss to trade through a single day's fog in a large city is something enormous, hardly less than that of a general holiday, with none of its advantages. To sum up a few of the chief arguments. Our citizens are allowing the air to be fouled, their lungs to be destroyed, their lives to be shortened, their property to be damaged, their purses to be impoverished, their race to be deteriorated, nay, the very light of day to be cut off from them by the present reckless output of smoke.

In the far-off future, when the dreams of the sanitary reformer shall have been fulfilled, the dweller in Cheapside may haply grow roses and columbine on his windowsill. The present is the age of science, and on its progress will largely depend any additions to the happiness of mankind. The coming race will have learnt how to live simply and to maintain a healthy balance betwixt body and mind. A paternal Government, freed from the anxiety of foreign brawls—for peace will reign amongst mankind—will place pure food and pure water within the reach of all, and will attend to the many problems concerned in bringing earth, air, fire, and water into the proper service of the community And when from the ashes of the Old World shall have arisen a New, fairer than that discovered by Columbus, its atmosphere will be pure as the untainted breezes of mid-ocean.

